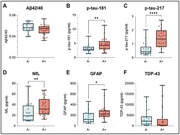# Exploring Blood‐Based Biomarkers in a Comparative Study of Amyloid‐Negative and Amyloid‐Positive Clinical Alzheimer's Syndrome

**DOI:** 10.1002/alz70856_101554

**Published:** 2025-12-25

**Authors:** Durjoy Lahiri, Jennifer G Cooper, Bruna Seixas Lima, Carlos Tyler Roncero, Cheryl L Wellington, Mari L DeMarco, Howard Chertkow

**Affiliations:** ^1^ Department of Medicine, Queen's University, Kingston, ON, Canada; ^2^ Translational Institute of Medicine, Queen's University, Kingston, ON, Canada; ^3^ Division of Neurology, Kingston Health Sciences Center, Kingston, ON, Canada; ^4^ University of British Columbia, Vancouver, BC, Canada; ^5^ Rotman Research Institute, Toronto, ON, Canada; ^6^ University of Toronto, Toronto, ON, Canada; ^7^ McGill University, Montreal, QC, Canada; ^8^ Providence Health Care, Vancouver, BC, Canada; ^9^ Canadian Consortium on Neurodegeneration in Aging (CCNA), Montreal, QC, Canada; ^10^ Kimel Family Centre for Brain Health and Wellness and Anne & Allan Bank Centre for Clinical Research Trials, Baycrest Health Sciences, Toronto, ON, Canada; ^11^ Division of Neurology, Department of Medicine, University of Toronto, Toronto, ON, Canada; ^12^ Baycrest and Rotman Research Institute, Toronto, ON, Canada; ^13^ Rotman Research Institute, Baycrest Health Sciences, Toronto, ON, Canada

## Abstract

**Background:**

It has been documented, 25‐33% of individuals clinically diagnosed with Alzheimer's syndrome, commonly referred to as the Alzheimer's phenotype, do not exhibit amyloid plaques in the brain upon autopsy. While non‐Alzheimer pathologies are suspected to contribute to misdiagnosis and cognitive decline observed in these patients, pathological distinctions between amyloid‐positive (Aβ+) and amyloid‐negative (Aβ‐) individuals remain poorly understood. Further investigation into these differences using blood‐based biomarkers may provide valuable insights into the underlying pathophysiology of the amyloid‐negative subgroup. Our objective was to compare blood‐based biomarkers between Aβ‐ and Aβ+ subgroups diagnosed with clinical Alzheimer's syndrome.

**Method:**

Participants were recruited from the screening phase of clinical trials investigating anti‐amyloid agents. A retrospective chart review was conducted to collect demographic, clinical, imaging, biomarker, and neuropsychological data. Amyloid‐beta (Aβ) status was assessed through cerebrospinal fluid (CSF) analysis using the Roche‐Elecsys β‐Amyloid (1‐42) CSF II assay or positron emission tomography (PET) imaging. Plasma samples were analyzed on the Quanterix Simoa HD‐X platform, employing neurology‐4‐plex‐E, *p*‐tau‐181, *p*‐tau‐217, and TDP‐43 assays. Statistical analyses were performed using the Mann‐Whitney U test for continuous variables and Fisher's exact test for categorical variables to compare group differences.

**Result:**

Of *n* = 45 patients, *n* = 25 (56%) were classified as Aβ+, and *n* = 20 (44%) were classified as Aβ‐. No significant differences were found between groups in terms of age, sex, duration of illness or cognitive presentation. Groupwise analysis revealed significantly elevated concentrations of phosphorylated tau (*p*‐tau) proteoforms in the Aβ+ group, including *p*‐tau 181 (4.32 vs 2.93 pg/mL, *p* = 0.0058) and *p*‐tau 217 (1.36 vs 0.46 pg/mL, *p* <0.0001), as well as glial fibrillary acidic protein (GFAP) (223 vs 123 pg/mL, *p* = 0.0265) and neurofilament light chain (NfL) (31.8 vs 19.1 pg/mL, *p* = 0.0068), relative to the Aβ‐ group. Although no significant differences were observed for Aβ42/40 (0.0548 vs 0.0567 pg/mL, *p* = 0.1466) or TDP‐43 (2219 vs 359 pg/mL, *p* = 0.0607), the Aβ‐ group exhibited markedly higher TDP‐43 concentrations compared to the Aβ+ group.

**Conclusion:**

These findings highlight distinct biomarker profiles between Aβ+ and Aβ‐ individuals, offering insights into the pathophysiology of clinical Alzheimer's syndrome.